# Depressive Symptoms in Multiple Sclerosis from an In Vivo Study with TBSS

**DOI:** 10.1155/2014/148465

**Published:** 2014-05-04

**Authors:** Yujuan Shen, Lijun Bai, Ying Gao, Fangyuan Cui, Zhongjian Tan, Yin Tao, Chuanzhu Sun, Li Zhou

**Affiliations:** ^1^Department of Neurology, Dongzhimen Hospital Affiliated to Beijing University of Chinese Medicine, Beijing 100700, China; ^2^The Key Laboratory of Biomedical Information Engineering, Ministry of Education, Department of Biomedical Engineering, School of Life Science and Technology, Xi'an Jiaotong University, Xi'an 710049, China; ^3^Department of MRI Scanning, Dongzhimen Hospital Affiliated to Beijing University of Chinese Medicine, Beijing 100700, China

## Abstract

Clinically significant depression can impact up to 50% of patients with multiple sclerosis (MS) over a course of their life time, which is associated with an increased morbidity and mortality. In our study, fifteen relapsing-remitting MS (RRMS) patients and 15 age- and gender-matched normal controls were included. Diffusion tensor imaging (DTI) was acquired by employing a single-shot echo planar imaging sequence on a 3.0 T MR scanner and fractional anisotropy (FA) was performed with tract-based spatial statistics (TBSS) approach. Finally, widespread WM and GM abnormalities were observed in RRMS patients. Moreover, the relationships between the depressive symptoms which can be measured by Hamilton depression rating scale (HAMD) as well as clinical disabilities measured by the expanded disability status scale (EDSS) and FA changes were listed. There was a positive relation between EDSS and the FA changes in the right inferior parietal lobule, while negative relation was located in the left anterior cingulate cortex and hippocampus. Also a positive relation between HAMD and FA changes was found in the right posterior middle cingulate gyrus, the right hippocampus, the left hypothalamus, the right precentral gyrus, and the posterior cingulate which demonstrated a link between the depressive symptoms and clinically relevant brain areas in RRMS patients.

## 1. Introduction

Multiple sclerosis (MS) is an autoimmune disease of the central nervous system (CNS) characterized by inflammation, demyelination, and neurodegeneration [1] and it is the most common cause of neurological disability among young adults, affecting approximately one in 1,000 individuals in Europe and North America [2]. Affective disorders are common and disabling conditions in MS. Clinically significant depression can affect up to 50% of patients with MS over the course of their lifetime and it is associated with an increased morbidity and mortality [3]. Also disability is a common symptom in MS patients [4]. The factors responsible for mood disturbances in MS are still controversial: a psychological reaction to a progressively disabling and unpredictable disease may be a relevant contributor while reactive mechanisms alone are probably not sufficient to account for the high prevalence and wide spectrum of depression.

Brain magnetic resonance imaging (MRI) lesions are highly associated with depression in MS [5, 6]. Neuroimaging studies in patients with MS have revealed associations between brain abnormalities and depression. The study by Pujol et al. [7] using axial spin echo sequence investigated three major anatomic divisions (basal, medial, and lateral) of the frontotemporal WM. They found that depressive score was significantly correlated with lesion load (LL) of the left accurate fasciculus region; in particular lesions in this area accounted for 17% of depressive score variance. Another MRI study including 95 consecutive MS patients, of which 19% of the patients met the criteria for depression, reported that the severity of depression was correlated with right frontal lobule and with right temporal brain atrophy; furthermore, T1 lesions in the superior parietal and superior frontal regions predicted depression in MS patients [8].

Ordinary MRI tests could sensitively display MS lesions; however they lack pathological specifications [9]. Researchers have proved that the concealing injuries existed in MS patients while the outcomes of ordinary MRI displayed normal [10] (i.e., the micropathology alteration exists and its DTI data changes also) [11, 12]. DTI enables the random diffusional motion of water molecules to be measured, thus providing metrics such as mean diffusivity (MD) and FA in order to allow quantification of the size and geometry of water-filled spaces [13] and provide complete pathology message of brain tissues. In the past few years, DTI studies have been widely applied to the CNS of MS and optical neuromyelitis, and they have demonstrated a powerful means to early diagnosis and patient's condition monitoring. 

Previous DTI studies generally adopted region of interest based analysis which needs specially prior information. Considering that MS may involve wide range of axonal degeneration, the whole brain analysis may be an optical method and lead to improved sensitivity and specificity to the disease and its related clinical impairments. Methods on the structural changes of WM fiber tracts in current research are mainly including hand-painted region of interest (ROI), voxel-based analysis (VBA), and TBSS, in which VBA is the most common research method. But for the anisotropic larger DTI image space, the registration accuracy is not high by VBA method which will lead to a certain difference based on the research results for the DTI images when different researchers are in view of the same kind of neurological diseases. However, TBSS analysis method aims at the main problem of image space registration rate, modifying from the registration rate algorithm, and makes the registration rate improved. So we chose TBSS as the analysis method. TBSS, adopting skeletonized processing ideas, projects individual fiber bundle FA value onto average FA bundle skeleton templates and accomplishes the justification of different subject fabric without taking standardization and smoothing [14] and thus significantly improves group comparison fidelity.

In the present study, we aimed at exploring global microstructural changes in MS and defining which changes are particularly affected by the disease such as the EDSS and HAMD scores and inquiring the relationships between FA values and HAMD as well as EDSS.

## 2. Materials and Methods

### 2.1. Subjects

In this study, we recruited the 15 patients who suffered from RRMS and were treated in Dongzhimen hospital in-patient or out-patient department from January 2012 to November 2013. All patients were in line with the revised McDonald criteria [15] and classification standard. The inclusion criteria were (1) participants were in the remission stage, who had no acute attack and did not have an exacerbation of their MS during the last month; (2) they were not currently taking any glucocorticosteroid medication; (3) their medication and treatment had no obvious adjustment recently and all of them had no history of serious psychiatric illness or neurologic disease other than MS; (4) Chinese was the primary language of all the participants; (5) the right-handed subjects according to the modified Edinburgh Handedness Questionnaire [16] were included. Those participants who had contraindications to MRI, poor quality of the images acquired, or showed one or more gadolinium-enhancing lesions (GEL) on baseline MRI were excluded to avoid effects of edema and inflammation on DTI measures [17]. Fifteen age- and sex-matched right-handed healthy subjects were used as a control group.

### 2.2. Clinical Assessments

For every participant, sex, age, onset age, disease duration, first onset symptoms, and recurrent symptoms variables were obtained while healthy controls' age and gender were also collected. A single neurologist assessed patients' disability using the EDSS [18] at the day of the neuropsychological assessment. All subjects were clinically evaluated by means of HAMD by themselves and two highly trained doctors on the same day prior to scanning. In this study, we used the HAMD 24 version and the criteria were as follows: total score *t* < 8 was divided into normal, 8–20 might suffer from depression, 20–35 was a depressive patient certainly, and >35 suffered from the severe depression.

### 2.3. MRI Acquisition

In the study, high-resolution brain MRI was acquired by using 2 pulse sequences on a 3T Signa scanner (Verio, Siemens AG, Erlangen, Germany) with an 8-channel head coil. The following sequences were acquired in a single session: (1) functional EPI oriented parallel to the AC-PC line and covering the whole brain to obtain sagittal sequence (repetition time (TR) = 2,000 ms, echo time (TE) = 30 ms, matrix = 64 mm × 64 mm, and field of view (FOV) = 225 mm × 225 mm), 36 slices, 3.5 mm thick, and no gap; (2) 3D magnetization-prepared rapid acquisition gradient echo (MPRAGE) sequence with 176 coronary, 1 mm slices, and 0 mm gap (TR = 2,700 ms, TE = 2.97 ms, flip angle = 7°, matrix = 256 mm × 256 mm, FOV = 250 mm × 250 mm, and voxel size= 1 mm × 1 mm × 1 mm); (3) T2-weighted image with a fast-spin echo sequence in the axial plane (TR = 6,000 ms, TE = 94 ms, matrix = 320 mm × 320 mm, and FOV = 220 mm × 220 mm), 25 slices, 4 mm thick, and no gap; (4) T2-Flair weighted image (TR = 8,800 ms, TE = 82 ms, flip angle = 150°, matrix = 256 mm × 256 mm, and FOV = 240 mm × 240 mm). DTI data acquisition was acquired with an axial single-shot echo planar spin echo sequence with 30 gradient directions (TR = 18,000 ms, TE = 94 ms, matrix = 160 mm × 160 mm, FOV = 256 mm × 256 mm, *b* = 0 and 1,000), 80 slices, 1.5 mm thick, and no gap. Image data processing was performed on a Linux workstation using Jim 5.0 software (Xinapse System, Leicester, UK; http://www.xinapse.com/), the Functional MRI of the Brain (FMRIB) software library (FSL) 4.1 package (FMRIB Image Analysis Group, Oxford, UK; http://www.fmrib.ox.ac.uk/fsl/), MATLAB 7.0 (MathWorks, Natick, Massachusetts, USA), and the Statistical Parametric Mapping 8 (SPM8) software (Wellcome Department of Cognitive Neurology, London, UK; http://www.fil.ion.ucl.ac.uk/spm/).

### 2.4. DTI Analysis

Diffusion data were preprocessed and analyzed using tools from the Oxford University Centre for FMRIB software library (FSL version 4.1). First, the b0 image of each subject was skull-stripped using the brain extraction tool. The data was corrected for subject motion and eddy-current induced geometrical distortions, and the diffusion sensitizing gradients (“bvecs”) were rotated to correct for motion. Using FDT, the diffusion tensor model was fit to the data, from which FA images were calculated.

For TBSS, all subjects' FA data was registered to a common space (the FA158 MNI space template) using a combination of affine and nonlinear registration. A mean FA image was created and eroded to a skeleton and threshold at FA > 0.25. Each subject's aligned FA data was then projected onto this skeleton and the resulting alignment-invariant representation of the central trajectory of WM pathways was used for voxel-wise statistical analysis (Randomize, 5000 permutations). The contrast TBI < controls was examined using threshold free cluster enhancement (TFCE), with correction for multiple comparisons at *P* < 0.05.

### 2.5. Statistical Analysis

The Statistical Package of Social Sciences (17.0; SPSS Inc., Chicago, IL) software was used to carry out the statistical analysis. All values were reported as mean ± standard deviation (SD) or median (range) as appropriate. Differences between groups were tested using paired *t*-test for continuous and categorical variables, respectively. Correlations among global DTI measures and between global MRI values and clinical scores (EDSS and HAMD) were analyzed by univariate analysis (Pearson's correlation coefficient) after correction for age, and results corrected for multiple comparisons were needed.

## 3. Results

### 3.1. Demographic and Clinical Characteristics

In our study, fifteen patients (4 males and 11 females) were obtained according to the inclusion and exclusion criteria, who were aged from 19 to 57 years (37.53 ± 11.57). The disease duration of these patients ranged from 2 to 34 years (7.27 ± 8.55), onset age was from 13 to 53 years (30 ± 11.62), and the median EDSS score was 2 (range from 0 to 4). We also recruited 15 sex- and age-matched healthy volunteers who were aged from 23 to 60 years (36.67 ± 12.60) and had no history of physical illness or psychiatric disorder, and their nervous system examination was normal as the control group. A paired *t*-test was served to assess between-group differences of age between controls and patients (*P* = 0.407 > 0.05).

### 3.2. Clinical Rating Scales

In our research, patients and healthy controls (HC) were valued clinically by EDSS and HAMD. EDSS scores in patients ranged from 0 to 4 (1.73 ±1.36), and in HC EDSS scores were all 0. HAMD scores in patients were from 1 to 35 (12.80 ± 11.620), while in HC they were from 0 to 14 (5.07 ± 3.918). Our study showed that there was a significant difference in HAMD score (*P* = 0.046 < 0.05) between patients and HC. And among the 15 patients there were 9 people whose HAMD score was greater than or equal to 8, accounting for 60%.

### 3.3. TBSS Analysis

Quantitative comparison for TBSS analysis demonstrated widespread statistically significant differences in FA values (*P* < 0.05, corrected for multiple comparisons), and FA values in all patients were lower compared with control subjects. Areas of reduced FA were seen widely in the GM and WM, such as the frontal lobe, limbic system, occipital lobe, temporal lobe, and parietal lobe. In particular, the main differences were located in bilateral corpus callosum, inferior parietal lobule, precentral gyrus, postcentral gyrus, superior frontal gyrus, cingulate gyrus, cerebellar lingual, declive, culmen, fastigium, dentate nucleus, parahippocampal gyrus, hippocampus, precuneus, basal ganglia, hypothalamus, insula, thalamus, fusiform gyrus, superior and transverse temporal gyrus, and the left middle temporal gyrus. Regional increases in the FA values of patients were not found. Compared with HC, the main lesions of the reduced FA value which has statistical differences and voxel were listed in Tables 1 and 2.

### 3.4. Correlation between Diffusion Parameters (FA) and Clinical Scores

Significant correlations were found between FA and EDSS in some lesions of WM and GM: the right inferior parietal lobule of WM (*r* = 0.6307, *P* = 0.0117), the left anterior cingulate (*r* = −0.5505, *P* = 0.0335), and hippocampus (*r* = −0.5143, *P* = 0.0498) of GM.

We also found the significant correlations between FA and HAMD in some lesions of WM and GM: the right posterior middle cingulate gyrus (*r* = 0.6265, *P* = 0.0124), hippocampus (*r* = 0.5742, *P* = 0.0252), and the left hypothalamus (*r* = 0.5357, *P* = 0.0396) of GM; the right precentral gyrus (*r* = 0.6575, *P* = 0.0077), cingulate gyrus (*r* = −0.5959, *P* = 0.091), and posterior cingulate (*r* = 0.5742, *P* = 0.0258) of WM.

**Figure 1:**
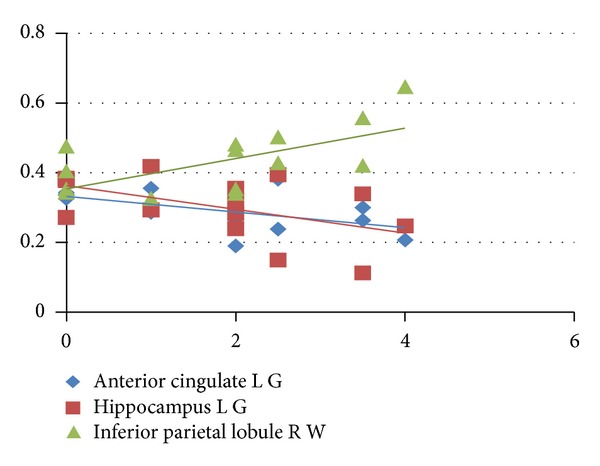
The correlation between EDSS and MRI lesion in GM and WM.

**Figure 2:**
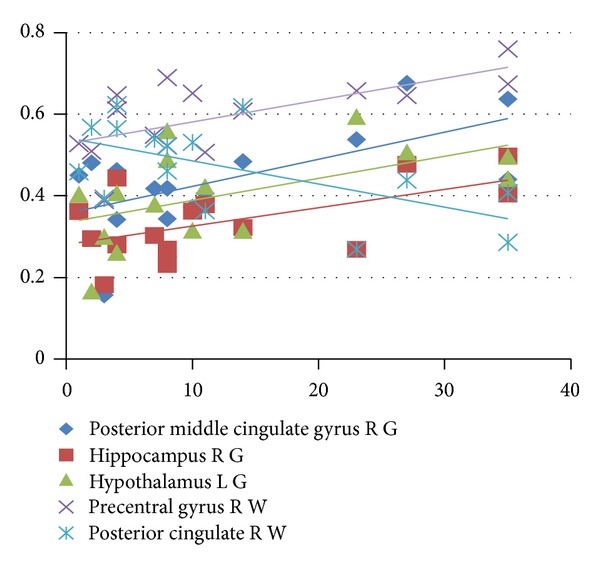
The correlation between HAMD and MRI lesion in GM and WM. R: right, L: left, G: GM, and W: WM.

*R* statistics (http://www.r-project.org/) analysis providing Spearman correlation coefficients values and their statistical significance were reported in Table 3 and shown in Figures 1 and 2.

## 4. Discussion

In our work, the patients' onset age was from 13 to 53 years and the sex ratio (female to male) was 2.75. This result suggested that MS tends to appear in the young and middle aged females which was consistent with most of the previous studies [19, 20]. The statistical difference of HAMD score (*P* = 0.046 < 0.05) between patients and HC was obvious, and among the 15 patients there were 9 people whose HAMD score was greater than or equal to 8, accounting for 60%. This result indicated that MS patients had a high incidence of depressive symptoms which was supported by the previous literature study [21].

DTI, as a new technology, which developed on the basis of diffusion weighted imaging (DWI) and could display brain WM fiber bundle and its direction in vivo noninvasively, is mainly used to evaluate the structural integrity of the microstructure, water molecules isotropic and anisotropic diffusion movement, and so forth. The results of DTI manifested the damage lesions mainly by the measures of FA values and decreased FA values could indicate a result of demyelination processes [22]. In the present study, we found that all the patients presented attenuated FA values when compared with HC. Similar results could be seen in some recent studies [23, 24]. Our data also confirmed that abnormalities in all the patients involved both WM and GM damage, which was consistent with many researches [13, 25, 26]. And our imaging data indicated that the lesions in WM of patients were mainly located in bilateral frontal lobe, limbic lobe, parietal lobe, occipital lobe, temporal lobe, corpus callosum, and sublobar while in GM the numerous lesions were primarily located in the limbic, sublobar, and cerebellum. In some previous researches, lesion was seen not only in the neocortex (especially in the cingulate cortex) [25, 27] but also in the GM of the thalamus, hypothalamus, cerebellum [27], basal ganglia [27, 28], and hippocampus [27, 29]. Audoin et al. [30] have reported that GM atrophy is associated with the bilateral insula, orbitofrontal cortices, internal and inferior temporal regions, thalamus, caudate nuclei, lenticular nuclei, cerebellum, and the posterior cingulate cortex. According to our result, it was roughly consistent with these previous studies.

However, most previous works adopted regional analysis, studying only certain parts of the brain, such as the normal appearing WM (NAWM) and GM (NAGM) [31], or the cerebellum [32], thalamus [33], and corpus callosum [34, 35]. Different from these reports, we studied WM and GM of the whole brain to find out the lesions by using a relatively new analysis method—TBSS. Hence, our research showed wider range of the lesions and this study could more fully state the distribution characteristics of the lesion site in the patients' brain DTI.

In this work, we adopted HAMD as evaluation index of psychological function and the relationships between FA value of the lesions in WM and GM of the entire brain and the HAMD scores in patients were explored. We investigated that HAMD scores were positively correlated with FA values in the left hypothalamus, right posterior middle cingulate gyrus and hippocampus of GM, the right precentral gyrus, and posterior cingulate of WM. These results told us that depressive symptoms were mainly negatively associated with the degree of demyelinating lesions in limbic system and frontal lobe, which had been reported in the previous paper [36]. Gobbi et al. [37] reported that depression in MS is linked to the atrophy of cortical regions located in the bilateral frontal lobes. Feinstein et al. [31] found that depressed subjects had a higher hypointense lesion volume in the right medial inferior frontal region, while having a smaller NAWM volume in the left superior frontal region and lower FA in the left anterior temporal NAWM and NAGM regions, respectively. The cause may be that frontal lobe and limbic system are relevant to human's affect, memory, and learning. Once these functions defected, depression would occur in patients. Besides, we also found the negative correlations between FA and HAMD in cingulate gyrus, which was never found before. The mechanism is unknown, and the ongoing compensatory cerebral process at work in the MS brain attempting to maintain an euthymic state which was found by functional MRI (fMRI) [38] may be correlated with it.

EDSS is regarded as the evaluation index of the neurologic deficits and many papers have studied the relationship between FA value of the lesions and EDSS score. Some suggested that there was no correlation [33, 39, 40], while some showed that there was a positive correlation between them: EDSS was positively correlated with FA value of the normal appear thalamus (*r* = 0.66, *P* = 0.045) [41], caudate (*r* = 0.444, *P* < 0.01), and thalamus (*r* = 0.362, *P* < 0.05) [42] and was slightly negatively correlated with the atrophy of the right cerebellum (*r* = −0.37, *P* = 0.0027) [30]. However, one paper showed that it was strongly negatively associated with some lesions (*r* = −0.82, *P* = 0.013) [23]. But the cause of the negative correlation between EDSS and the degree of demyelination still needs further investigations. For the pathological changes in this relationship, according to Tedeschi et al. [43] and Hofstetter et al. [44], GM atrophy is associated with MS clinical disability. Routinely detectable cortical lesions are related to physical disability [45]. And Ciccarelli et al. [46] have reported that, in patients with RRMS, there was a strong correlation between EDSS score and FA in both supratentorial and infratentorial NAWM. Gorgoraptis et al. [39] found that smaller paracentral cortex volume was associated with worse walking ability, as measured by the TWT. One research showed that either the NAWM FA or the GM volume in each of these regions correlated with disability [47, 48]. Studies from DTI features of RRMS patients show that GM atrophy is a better indicator of disability progression than WM atrophy or accumulation of lesion burden [49–52]. In our study, EDSS is strongly correlated with FA value in the right inferior parietal lobule of WM positively and the left anterior cingulate and hippocampus of GM negatively in patients. Anterior cingulate cortex (ACC), including Brodmann 24, 25, and 32 area, which is located in the medial area of frontal lobe, can monitor the ongoing goal orientation behavior, provide signals in response to conflict or mistakes, and allocate the attentive resources effectively in related brain regions according to the requirements of the current task processing, and therefore it may be a senior regulatory structure in the executive function neural network [53], while hippocampus is responsible for learning and memory. Considering the functions of the damage regions in human, we all agreed that once these functions disappear this may lead to clinical disability in patients with MS.

However, our study has some limitations. First, this research only recruited the MS patients who were in remission and did not include patients who were in the acute stage as a control group for the related pathology study. Second, we conducted a cross-sectional study only, not for long-time follow-up observations. Third, in our work some appearances cannot be explained just with our present knowledge and findings. For instance, EDSS was negatively correlated with the degree of demyelination in the right inferior parietal lobule of WM and the further causes of the positive relationship between HAMD and FA in the lesions. For further study, we will expand the scope of the study population, conduct longitudinal observation on the basis of the study, and further analyze the relationships between the lesions and clinical scores.

## 5. Conclusions

In conclusion, our study used TBSS analysis method for the whole brain DTI of RRMS patients who were in remission and a large amount of information was provided for multiple areas of the brain GM and WM in pathological changes. The characteristics of the various lesion areas and the relationships between the clinical scores of MS patients were discussed in this paper, providing the possible mechanisms for the pathogenesis of MS. In addition, we had shown that GM damage could explain clinical depression and disability better than WM. These findings were important for our understanding of MS and for future clinical trial design. And TBSS can be useful in future studies with other MS patient samples to provide an unbiased localization of damage and generate location-specific hypotheses.

## Figures and Tables

**Table 1:** The areas of FA value significantly reduced in GM by TBSS analysis.

		Talairach			*t* value	*V* mm^3^
		*X*	*Y*	*Z*		
Limbic system						
ACG	L	−3	31	15	3.846	65
BA24	R	3	26	19	5.182	159
ACG	L	−3	4	−6	3.543	44
BA25	R	7	16	−8	3.238	45
PCG	L	14	−50	8	3.135	122
BA29/30	R	−5	−60	9	2.002	39
MCG	L	−20	−16	38	2.999	113
BA24	R	18	−3	45	4.299	250
PMCG	L	−18	−43	28	3.307	62
BA31	R	19	−43	37	4.761	102
Amygdala	L	−19	−6	−19	3.355	48
	R	19	−6	−18	2.113	81
Hippocampus	L	−30	−36	−6	2.948	35
	R	33	−14	−18	3.682	97
Subcortical						
Putamen	L	−22	−9	9	4.632	217
	R	23	−7	11	3.981	200
Thalamus	L	−5	−9	13	3.947	143
Cerebellum						
CL	L	−4	−46	−15	4.041	60
	R	3	−48	−18	2.968	32
Culmen	L	−1	−54	−14	4.073	1204
	R	14	−41	−18	4.317	894
Declive	L	−1	−55	−14	3.875	363
	R	3	−55	−11	4.160	152

**Table 2:** The areas of FA value significantly reduced in WM by TBSS analysis.

		Talairach			*t* value	*V* mm^3^
		*X*	*Y*	*Z*		
Limbic system						
ACG	L	−7	24	−4	4.655	158
BA24	R	15	18	23	4.275	318
CG	L	−15	−33	35	5.154	1561
	R	19	−28	34	4.884	1798
PHG	L	−22	−18	−13	4.989	663
BA28/36	R	19	−41	2	5.884	654
PCG	L	−11	−56	6	2.974	125
BA29/30	R	14	−53	14	4.149	253
Subcortical						
CC	IH	−1	−16	24	2.889	63
	L	−3	13	20	3.917	1180
	R	13	−29	25	6.924	1068
Insula	L	−38	−43	20	3.899	123
BA13	R	39	−41	19	4.562	107
Frontal lobe						
CG	L	−17	15	36	3.727	38
PrG	L	−25	−17	50	2.299	30
BA4	R	37	−10	26	2.836	55
CC	L	−11	20	18	3.835	207
	R	13	19	20	3.922	146
Parietal						
IPL	L	−42	−42	25	3.559	135
	R	38	−42	26	4.286	110
Precuneus	L	−21	−59	32	4.402	435
BA31	R	19	−42	44	5.347	271

BA: Brodmann area; ACG: anterior cingulate gyrus; CG: cingulate gyrus; PCG: posterior cingulate gyrus; MCG: middle cingulate gyrus; PMCG: posterior middle cingulate gyrus; CC: corpus callosum; IPL: inferior parietal lobule; PrG: precentral gyrus; DLPFC: dorsolateral prefrontal cortex; AG: angular gyrus; CL: cerebellar lingual; IH: interhemispheric; PHG: parahippocampal gyrus.

**Table 3:** Significant correlations (Spearman correlation coefficients) between diffusion parameters (FA) and clinical scores.

CRS and correlated lesions		*R* value	*P* value
EDSS (GM)			
ACC	L	−0.5505*	0.0335
Hippocampus	L	−0.5143*	0.0498
EDSS (WM)			
IPL		0.6307*	0.0117
HAMD (GM)			
pMCC	R	0.6265*	0.0124
Hippocampus	R	0.5742*	0.0252
Hypothalamus	L	0.5357*	0.0396
HAMD (WM)			
PrG	R	0.6575*	0.0077
CG	R	−0.5959	0.0910
PCC	R	0.5724*	0.0258

*Significant correlation, *P* < 0.05.

R: right, L: left, CRS: clinical rating scales, ACC: anterior cingulate cortex, IPL: inferior parietal lobule, pMCC: posterior middle cingulate cortex, PrG: precentral gyrus, CG: cingulate gyrus, and PCC: posterior cingulate cortex.
